# Splenic marginal zone lymphoma treated with laparoscopic splenectomy: A case report

**DOI:** 10.1016/j.ijscr.2019.11.008

**Published:** 2019-11-09

**Authors:** Ryota Koyama, Nozomi Minagawa, Yoshiaki Maeda, Toshiki Shinohara, Tomonori Hamada

**Affiliations:** Department of Gastrointestinal Surgery, Hokkaido Cancer Center, Sapporo, Japan

**Keywords:** Malignant lymphoma, Splenic marginal zone lymphoma, Laparoscopic splenectomy

## Abstract

**Introduction:**

Splenic marginal zone lymphoma (SMZL) is rare subtype of malignant lymphoma that is classified as a low-grade B cell lymphoma. Splenectomy is usually chosen for both diagnosis and treatment, which often leads to the resolution of abdominal symptoms and cytopenia.

**Case presentation:**

We presented the case of a 73-year-old woman who complained of spontaneous perspiration and fatigue. She was referred to our hospital for further treatment of enlarged intraabdominal lymph nodes and splenomegaly. The level of soluble Interleukin-2 receptor was elevated, and malignant lymphoma was suspected. Endoscopic ultrasound fine-needle biopsy of intraabdominal lymph node revealed B cell lymphoma. For a definitive diagnosis, as well as treatment, laparoscopic splenectomy was successfully performed. The histopathological finding was compatible with SMZL. The patient was discharged without any complications and observed to have no recurrence at eight months postoperatively.

**Conclusion:**

Patients with SMZL is usually expected to have good prognosis with splenectomy. However, careful follow-up is required for aggressive transformation can occur and result in worse prognosis.

## Highlights

• The authors present a case of splenic marginal zone lymphoma (SMZL), which was successfully treated with laparoscopic splenectomy.
• SMZL is a rare subtype of indolent B cell lymphoma, and good prognosis is expected by splenectomy alone.
• Laparoscopic splenectomy is safe and feasible with splenic artery embolization preoperatively, especially in cases with large splenomegaly as seen in our case.
• Careful postoperative follow-up is required since some patients develop aggressive transformation, and result in worse prognosis.

## 1 Introduction

Splenic marginal zone lymphoma (SMZL) is an uncommon subtype of B cell lymphoma, in which the tumorous lymphocytes proliferate in the form of a nodular architecture in the spleen. The World Health Organization classification defines SMZL as a subtype of marginal zone lymphoma [1]. It is usually a slow-growing tumor, but transformation to a high-grade lymphoma has been shown to occur in a small fraction of patients [2,3]; therefore, careful follow-up according to the predicted risk is warranted. The treatment of choice for SMZL has been splenectomy, but novel treatment options, such as the anti-CD20 antibody rituximab, is currently being considered [4]. We report the features of this case along with a review of the literature. This work has been reported in line with the SCARE criteria [5].

## 2 Presentation of case

A 73-year-old woman initially complained of perspiration and fatigue for 10 months prior to visiting another hospital, where abdominal ultrasound was done and detected multiple enlarged intraabdominal lymph nodes and splenomegaly. With an elevated level of soluble interleukin-2 receptor, the preliminary diagnosis was malignant lymphoma. She was referred to our hospital for further evaluation and treatment.

The patient was asymptomatic on admission. Her height was 153 cm and her weight was 54 kg. Her blood pressure was 142/73 mmHg, heart rate was 73 beats/min, and body temperature was 36.4 °C. The abdomen was soft and flat, without tenderness. The spleen was palpable within a length of two fingers in the left hypochondriac region. Laboratory study results showed slightly elevated C-reactive protein (0.92 mg/dL), but the other serum chemistry results were within normal limits. Complete blood count showed slight anemia (11.2 g/dL) and thrombocytopenia (114000/μL), but the white blood cell count was normal (3340/μL). The soluble interleukin-2 receptor level was high (4483 U/mL). Antihepatitis C virus antigen was negative.

**Fig. 1:**
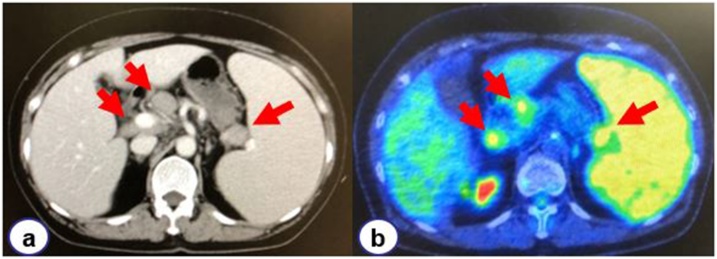
Computed tomography and PET findings. A splenomegaly, and multiple intraabdominal swollen lymph nodes were detected at splenic hilum, hepatoduodenal ligament, and along common hepatic artery (a). PET showed diffusely enhanced uptake in the spleen, intraabdominal lymph nodes and splenic hilum (b).

Contrast-enhanced abdominal computed tomography (CT) revealed splenomegaly with multiple swollen intraabdominal lymph nodes in the splenic hilum, hepatoduodenal ligament, and along the common hepatic artery (Fig. 1). Whole-body positron emission tomography/ CT showed diffusely enhanced uptake in the spleen [maximum standard uptake value (SUVmax) 4.53], which was higher than that in the liver, and enhanced uptake in the swollen intraabdominal lymph nodes (SUVmax 3.08–3.56) (Fig. 1). The lymph nodes in the splenic hilum had an SUVmax of 4.28.

Because the swollen lymph node in the splenic hilum was accessible by Endoscopic ultrasound fine-needle biopsy, histopathological diagnosis was successfully obtained. The specimen contained several small- to normal-sized homogeneous lymphoid cells. Immunohistochemistry of these cells was positive for CD20, which is characteristic of B cells. In addition, only few small T-cells that were positive for CD3/CD5 were found. Taken together, the final preoperative differential diagnoses included follicular lymphoma and SMZL. She was referred to our department for splenectomy to make a definitive diagnosis and possible simultaneous treatment, because splenectomy alone can resolve the symptoms and SMZL itself. To avoid massive hemorrhage during surgery and enable successful laparoscopic splenectomy (LS), splenic artery embolization (SAE) was performed one day before the surgery.

**Fig. 2:**
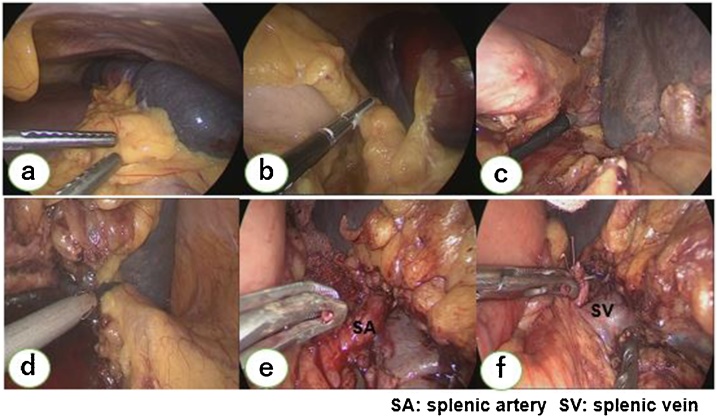
Intraoperative findings. The spleen showed partial ischemic change due to the splenic artery embolization (a). The gastrosplenic ligament was cut (b), and superior pole of the spleen was detached using laparoscopic coagulating shears (c). The splenocolic ligament was then dissected (d). At the splenic hilum, the splenic artery and vein were separately clipped and cut (e, f). The spleen became free from fixation to the adjacent organs and mobile by dissecting it from laterally.

The surgery was performed by five-port system. Intraoperatively, there were no intraperitoneal dissemination and ascites. The spleen showed partial ischemic changes due to the SAE. The gastrosplenic ligament was dissected, and, using laparoscopic coagulating shears, the upper pole of the spleen was detached (Fig. 2), followed by dissection of the splenocolic ligament. The splenic artery and vein in the splenic hilum were clipped and cut separately. The spleen was mobilized by dissecting it from the lateral site. A slight extension of the umbilical midline incision was required, in order to extract the enlarged spleen from the abdominal cavity. The operation time was 7 h and 10 min, and the blood loss was 752 mL, without the need for transfusion.

**Fig. 3:**
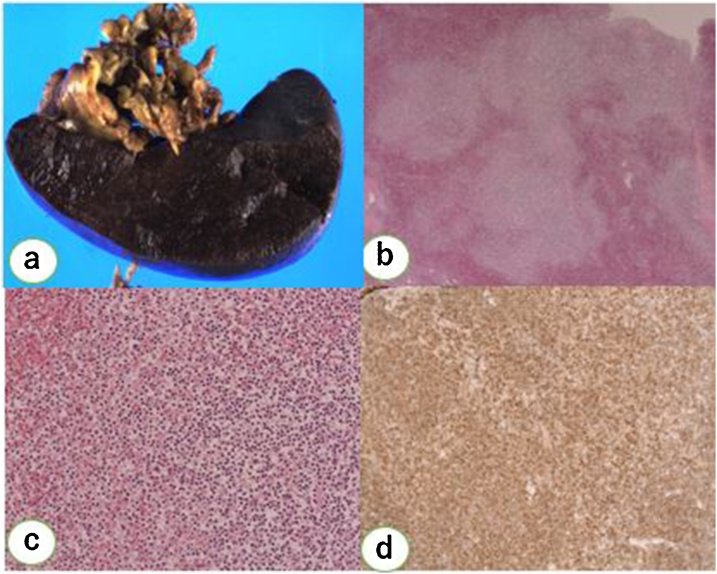
The resected specimen and histopathological findings. Macroscopically, the cut surface of resected spleen appeared dark reddish with white granular change (a). Histopathology showed small to medium sized lymphoid cells proliferating in nodules replacing splenic white pulp The proliferating cells resemble monocytoid B cells with diffuse infiltration into the splenic red pulp (b: H.E. ×20, c: ×100). Immunohistochemistry showed the tumor cells were positive for CD20 (d), which is known for B cell feature.

Macroscopically, the resected spleen appeared dark reddish with whitish granular changes on the cut surface and weighed 1700 g (Fig. 3). Histopathology showed small- to medium-sized lymphoid cells proliferating in nodules and replacing the splenic white pulp. The germinal center was obscure. The proliferating cells resembled monocytoid B cells and diffusely infiltrated the splenic red pulp. Immunohistochemistry was positive for CD20, which is a known B cell feature (Fig. 3), and negative for CD10 and BCL6. Histopathology also confirmed infiltration into the lymph nodes in the splenic hilum. The patient was discharged on postoperative day 15 without any complications. She remained free of relapse at eight months after surgery without additional treatment.

## 3 Discussion

SMZL is a rare form of indolent B cell lymphoma that accounts for <2 % of cases of non-Hodgkin’s lymphoma. SMZL affects the spleen, bone marrow, and peripheral blood [1].

Patients with SMZL presents with symptomatic splenomegaly and cytopenia in the latter course of the disease. Moreover, preoperative diagnosis has often been difficult, especially in the earlier stage, because of the absence of specific symptoms and findings on laboratory and imaging studies. Only histopathological examination of the spleen is required for a definitive diagnosis of SMZL. Patients with SMZL have been known to maintain remission for years after splenectomy alone. Therefore, splenectomy is usually chosen for both diagnosis and treatment [6]. In our case, the preoperative differential diagnoses were narrowed down to either follicular lymphoma or SMZL, based on the specimen obtained by EUS-FNA of the enlarged splenic hilar lymph nodes; therefore, EUS-FNA might be useful when there is a proper target lesion [7].

In SMZL, splenomegaly is usually seen, and a case with more than 2000 g of splenic weight has been reported [8]. On histopathology, lymphoma cells usually replace the white pulp or proliferate in the marginal zone of the white pulp, and patchy or diffuse infiltration of tumor cells in the splenic sinuses of the red pulp may also be observed. In peripheral blood cells, villous lymphocytes with round nuclei, condensed chromatin, and basophilic cytoplasm with polar short villi are characteristic of SMZL and are required to differentiate SMZL from hairy cell leukemia [9,10]. The immunophenotype of SMZL is usually positivity for CD20, D79a, Bcl2, and surface immunoglobulin M (IgM) and negativity for CD5, CD10, BCL6, and CD43, and CD103. However, these findings are nonspecific; therefore, flow cytometry and immunohistochemistry have been mainly utilized to exclude the other types of lymphomas [1].

Most of the SMZL cases reported had indolent clinical courses. However, clinicians must monitor for transformation into a more aggressive type of lymphoma, such as diffuse large cell lymphoma, which can occur in a small number of SMZL cases and has worse prognosis [2,3]. Development into high-grade lymphoma involves the bone marrow, liver, spleen, lymph nodes, and central nervous system [11,12].

Consensus guidelines recommended treatment of only selected patients, such those with symptomatic splenomegaly, cytopenia, systemic symptoms, or aggressive nodal disease [13,14]. Splenectomy has been the management of choice for the definitive diagnosis and treatment of SMZL [15]. On the other hand, administration of the anti-CD20 monoclonal antibody rituximab, alone or combined with chemotherapy, has emerged as a treatment option [16]. Recently, hepatitis C virus infection has been reported to be related with the etiology of SMZL, thereby, prompting ways for the novel treatment options that include antiviral agents [17].

As chosen for our case, splenectomy alone contributes to the resolution of abdominal symptoms due to splenomegaly. Importantly, it may also resolve cytopenia and remission for many years [6]. As a surgical approach, we chose LS for its advantages [18], such as decreased blood loss and fewer complications, compared with those of open splenectomy. According to the 2008 European guidelines, LS for massive splenomegaly (i.e., spleen size >20 cm) is generally not recommended. In our case, although the spleen size was 16 cm, we decided to perform SAE preoperatively to reduce the risk of needing transfusion [19]. To avoid SAE-related complications, such as pancreatitis and acute gastric ulcer, it was performed one day prior to the operation [20]. Fortunately, we successfully resected the large spleen laparoscopically and without the need for transfusion.

Among the proposed prognostic factors of SMZL, our patient had anemia, and advanced age [21]. The others are reported to be leukocytosis, lymphocytosis, thrombocytopenia, monoclonal component, β2 microglobulin, performance status of ≥2, incomplete response, nonhematopoietic site involvement, and diffuse pattern of bone marrow infiltration [6,21,22]. Currently, the patient is in remission, with no additional treatment postoperatively.

## 4 Conclusion

SMZL is usually an indolent type of lymphoma and is expected to have good prognosis, with appropriate treatment. However, in some patient populations, aggressive transformation can occur and result in worse prognosis. The need to explore the clinical characteristics of patients with SMZL is required in the future.

## Declaration of Competing Interest

The authors (RK, NM, YM, TS & TH) declare no conflicts of interests or disclosures.

## Sources of funding

This work received no funding.

## Ethical approval

This study is exempt from ethical approval in our institution.

## Consent

Written informed consent was obtained from the patient for publication of this case report and accompanying images. A copy of the written consent is available for review by the Editor-in-Chief of this journal on request.

## Author’s contribution

RK is the primary investigator and contributed to conceptualization, data collection and drafting the manuscript. All authors have read and approved this manuscript for publication.

## Registration of research studies

The name of the registry is research registry. The unique identifying number (UIN) is researchregistry5201.

## Guarantor

Ryota Koyama.

Tomonori Hamada.

## Provenance and peer review

Not commissioned, externally peer-reviewed.
